# Measuring a panic buying behavior: the role of awareness, demographic factors, development, and verification

**DOI:** 10.1016/j.heliyon.2022.e09372

**Published:** 2022-05-05

**Authors:** Othman A. Alfuqaha, Dua'a A. Aladwan, Yazan Al Thaher, Fadwa N. Alhalaiqa

**Affiliations:** a Jordan University Hospital, The University of Jordan, Amman, 11942, Jordan; b Faculty of Arts and Sciences, Applied Sciences Private University, Amman, 11931, Jordan; c Faculty of Pharmacy, Philadelphia University, Amman, 19392, Jordan; d Faculty of Nursing, Philadelphia University, Amman, 19392, Jordan

**Keywords:** Panic-buying behavior scale, Awareness, COVID-19, Development

## Abstract

In this study, a new panic buying behavior (PBB) and awareness scales were established and verified during the Coronavirus Disease 2019 (COVID-19) pandemic. The PBB scales measured the relationship between people's awareness about COVID-19 and PBB. Moreover, this study investigated the potential association of some demographic factors with PBB. The online descriptive cross-sectional survey was collected from 429 Jordanians who were selected using the snowball sampling technique. The online survey started from April 1st to April 10th, 2020. Pearson correlation coefficient, one-way analysis of variance, independent sample t-test, and linear regression were used in this study. Findings indicated that both established scales were valid and reliable for achieving the required level of validity and reliability. In the current study, participants' awareness of COVID-19 was high; but, their PBB was low. Single and young individuals were positively associated with PBB. Awareness about COVID-19 and age were found to be the main predictors of PBB. This study concluded that the higher the awareness level about COVID-19, the lower PBB. High PBB may affect the world economy, highlighting the importance of COVID-19 awareness.

## 1 Introduction

Coronavirus disease 2019 pandemic (COVID-19) has spread rapidly across the world, instilling fear and insecurity, as well as loss of control and stability in people (Arafat et al., 2021b; Livingston and Bucher, 2020). Also, COVID-19 pandemic has altered our daily living (restrictions on mobility, travel, and social contact). Consequently, global economies and industries were affected negatively (Loxton et al., 2020; Zvolensky et al., 2020). Since the pandemic occurred unexpectedly, most countries experienced panic buying behavior (PBB) to certain levels (Arafat et al., 2020b).

PBB can be defined as purchasing larger-than-normal quantities of everything from what a person needs (bread, rice, vegetables, fruits, toilet paper, and disinfecting products) (Arafat et al., 2020a). This behavior could be influenced by variety of factors, including socio-cultural norms, moral instructions, and personality types (Arafat et al., 2021a). Also, it is associated with panic feelings, high anxiety, anger levels, lower logical reactions, and emotional reactions (Yuen et al., 2020). Furthermore, fear caused by social media leads to panic buying as a mediating factor (Ahmed et al., 2020). PBB is a self-preservation and rational behavior that limits the exposure between individuals during the pandemic. Therefore, PBB is essential in crises to certain limits to hoard goods for a few days (Arafat et al., 2021b).

With frequent lockdown, individuals may experience fear about the future because of the pandemic; hence, they are involved in PBB (INSEAD, 2020). This fear is caused by uncertainty and hard situation brought by the pandemic, possibility of shortage for essential products, and low levels of trust in the government to handle the situation (Ahmed et al., 2020). Consequently, hundreds of people may have to wait several hours in lines in front of shopping centers. Thus, this behavior increases the risk of being exposed to COVID-19. According to the World Health Organization (WHO), crowded areas such as shopping malls accelerate the spread of COVID-19 (WHO, 2020). In Jordan, the number of new cases and deaths from COVID-19 was increasing rapidly, particularly among patients with comorbidities (WHO, 2020). Therefore, governments have imposed lockdowns numerous times in order to contain the spread of the COVID-19 pandemic. Consequently, people's PBB increased, leading them to more shopping to meet their expected needs. This behavior can lead to serious adverse events, causing the spread of COVID-19 and increasing the burden on health care systems worldwide. However, increasing individual awareness about COVID-19 plays a vital role in reducing negative feelings such as fear, anger, panic, and anxiety (Harlianty et al., 2020).

In general, awareness has the potential to provide an antidote to such behavior. It is important to be aware about the importance of personal protective equipment, the importance of hand hygiene and social distancing. Awareness is an effective way to avoid frequent COVID-19 exposure, allowing people to reject unwanted panic behavior, managing their anxiety level, and keeping themselves and their families safe during the spread of the COVID-19 pandemic (Mukhlis et al., 2022). Awareness about personal protective equipment (e.g. masks, gloves) and frequent hand hygiene continues to be the best way to prevent the spread of COVID-19 (Ton et al., 2020). Moreover, social distancing awareness is the predominant way to reduce the risk of COVID-19 transmission (Sun and Zhai, 2020). There are many sources for news and information about COVID-19, the majority of which are invalid, leading to rumors and panic behaviors (Hou et al., 2020). Only official information from authorities can help raise public awareness (Hou et al., 2020).

### 1.1 Literature review

Previous studies showed that people with awareness reported less fear, panic and anxiety feelings and behaviors, and have better ability to manage and understand the information about the pandemic spreading (Lee and Lee, 2019; Mishra et al., 2016). Consequently, it is essential to evaluate the relationship between the level of awareness regarding COVID-19 among people (personal protective precaution, hand sterilization, rumors, and social distancing) and PBB.

Many demographic factors are associated with an increased level of fear, panic, anxiety, and stress, such as being female (Harlianty et al., 2020; Lee and Lee, 2019; Mishra et al., 2016). Previous studies showed that employment status, experience, age, income, and marital status were associated with PBB (Li et al., 2021; Wang et al., 2018). However, the potential association between such several socio-demographic factors and PBB has not been investigated properly (Leung et al., 2021).

Consumers responses to the COVID-19 pandemic are different across the world (Andersen et al., 2020; Valaskova et al., 2021). Consumers have experienced different norms and attitudes in terms of preparing for the pandemic and type of purchased goods (Naeem, 2021). For example, consumers in developing countries (e.g. Jordan and Lebanon) stocked up essential foods such as bread, rice, and sugar (The Jordan Times, 2020). Whereas, consumers in developed countries (e.g. United States and Australia) stocked up toilet paper, hand sanitizer, and canned food (Stratton, 2021).

The potential explanations for these differences are related to environmental factors, cultural factors, and personal factors, as well as the economic repercussions in developing countries. Developing countries were not prepared for the crisis compared to developed countries. Specifically, the attitude of Jordanians consumer behavior was expressed as waiting several hours in lines at groceries and bakeries, searching for food with long shelf-life. Therefore, studying these differences will provide sufficient information by prompting us to explore Jordanians consumer behavior during the pandemic. To the best of our knowledge, no previous studies measured PBB and used a valid and reliable scale among developing Arab countries. In a developing non-Arab country, there was only one study developed and validated a panic-buying scale among 393 participants in Brazil (Lins and Aquino, 2020). In fact, different countries require tailored scales according to the cultural differences between populations at different economic levels. This study aims to highlight the importance of COVID-19 awareness, providing Jordanians with valuable evidence to limit PBB.

This study will explore Jordanians’ PBB during COVID-19 pandemic and highlight the importance of public awareness toward any crises in the future. The organizational and methodological process of this study was carried out based on three steps. In the first step, we established a new PBB and awareness scales by following theoretical and methodological substantiations. In the second step, we distributed the valid scales among Jordanians using an online survey. In the last step, we looked into Jordanians PBB and their awareness toward COVID-19. Finally, we investigated the association between PBB and demographic factors (gender, marital status, age, income, and educational level).

## 2 Materials and methods

### 2.1 Study design

A descriptive cross-sectional design was used to investigate the Jordanian's perception toward PBB and COVID-19 awareness by distributing an online reported survey through social media. Notably, other recent studies used the same cross-sectional design (Di Crosta et al., 2021; Helisz et al., 2021).

### 2.2 Participant and procedure

We used an online survey due to lockdown periods. We invited participants who met the inclusion criteria which are; Jordanians with a minimum age of 18, who can read Arabic language, and have access to social media. The online reported survey via Microsoft teams started from April 1st to April 10th, 2020. Also, the survey was posted on social media through Facebook, Messenger, and WhatsApp. The snowball sampling technique was used by asking the participants to share the selected online reported survey with their acquaintances (Ghaljaie et al., 2017).

Selected social media users we invited to distribute the online survey on their relatives, to limit potential source of bias among participants. Furthermore, we focused on the social media groups who can give adequate information. In this regard, we selected the most trusted popular Jordanian social media groups that included large audience with diverse demographic factors. However, we included specific questions based on selected demographic factors (nationality, age above 18); when participants showed wrong answer, they were excluded from the survey. The online survey took an average of five minutes to complete. Finally, participants' rights, including confidentiality, privacy, withdrawal from the study, were confirmed in the survey's first paragraph. The last sentence confirmed that completing the survey represents an informed consent. A total of 500 participants were needed to have the required sample size (Hair et al., 2014a, Hair et al., 2014b). Therefore, 500 participants filled the online reported survey. We excluded a total of 71 participants due to unfinished and outlier's data, leaving a sample size on 429.

Participants were asked to fill three sections: consent form, demographic factors form (gender, marital status, age, income, and educational level) and scales (established PBB and awareness scales), which are described below.

### 2.3 Study instruments

For the purpose of the current study, we developed PBB and awareness scales based on theoretical and methodological substantiations. Theoretical substantiation for the developed items was done by reviewing literature and pre-existing scales. In this regard, Jaber et al. developed a new awareness scale during COVID-19 in the Middle East (Jaber et al., 2021). While, a new validated panic-buying scale was developed in Brazil (Lins and Aquino, 2020). In respect with theoretical analysis, we asked Jordanian experts (i.e., 2 specialized in psychology, 2 specialized in psychiatry, 2 specialized in counseling, and 2 specialized in economy), to make amendments, omissions, and additions to both scales. At the same time, the importance of the scales was assessed by 3 Point-Likert scale (appropriate/not appropriate, suitable/not suitable for local needs, and belonging/not belonging). Finally, 13-items on the PBB scale and 10-items were developed. Some examples of PBB items were included “I lost control of buying a huge amount of all home necessities” and “I queued for a long time to meet my basic and non-basic needs”. The awareness scale involved 10 items to measure the perceived level of awareness in terms of personal protective equipment, government policy, behavioral response, attitudes towards the situation, and social distancing. Details of items included in the PBB and awareness scales are included in appendix Table S1. Methodological substantiation was assessed by psychometric analysis, which are described in the following section.

These scales were measured on a 4-point Likert type scale. PBB and awareness scales were rated as follows: 1 "Strongly Disagree," 2 "Disagree," 3 "Agree," 4 "Strongly Agree." Cutoff point was calculated based on Eq. (1):

(1) Cutoff point= (upper score – lower score)/levels

In Eq. (1), the overall perception levels were determined as a low average score ranged between (1.00–2.50), and the high average score ranged between (2.51–4.00).

### 2.4 Ethical approval

Ethical approval was obtained from the institutional review board. The declaration of Helsinki 1975 was followed in all study conduction steps, and all rights of the participants were confirmed. The participants were identified through the data collection date, and the data was stored in the principal investigator's computer. The principal investigator accessed the data, who kept it in the computer, which was password-protected. Therefore, their confidentiality was maintained.

### 2.5 Statistical analysis

Statistical analysis was performed using a statistical package for social science (SPSS V.23). Explanatory and confirmatory factor analyses (EFA and CFA, respectively) were conducted to test the validity and reliability of the selected scales. Content validity was checked by sending the PBB and awareness scales to 8 arbitrators, including professors specialized in psychology, psychiatry, nursing, medicine, and economy. These arbitrators were asked to provide their views and feedback on each item for PBB and awareness scales. According to Lawshe's content validity ratio Table, a percentage of 0.75 is a minimum score for accepting content validity which is needed from 8 arbitrators for each item (Polit et al., 2007). Table 1 shows the accepted values for item-level content validity ratio with different numbers of arbitrators and agreements.

**Table 1:** Accepted values of item-level content validity ratio with different numbers of arbitrators and agreement.

Number of experts	Item-level content validity ratio	Judgment
3	0.67	Fair
4	0.75	Good
5	0.80	Excellent
6	0.83	Excellent
7	0.71	Good
8	0.75	Good
9	0.78	Excellent

*Note.* Lawshe's content validity ratio.

Before analysis, several assumptions were checked regarding normality, multicollinearity, outliers, and reasonable highly correlations between the items. For the execution of EFA, we used the following criteria: Eigenvalues more than 1 considered factor (Onatski, 2010). Sampling adequacy assessed by using the Kaiser-Meyer-Olken (KMO) test and Bartlett's test of sphericity. A KMO value greater than 0.70 was considered sufficient, and the chi-square (*χ2)* value was significant (Kaiser, 1970). Furthermore, Varimax rotation with equal or above 0.40 was considered sufficient (Awang, 2015). CFA was measured using the analysis of moment structures (AMOS V.26). Cross-loading for every item on this scale should be greater than 0.40 (Awang, 2015). Also, fit indices were measured as follows: relative chi-square was less than 3 (MacCallum et al., 1996). Goodness of fit index value, adjusted goodness of fit index value, comparative fit index value, increment fit index value, and Tucker-Lewis index values were above 0.90 suggesting an acceptable model fit (Awang, 2015; Bentler, 1990). Finally, root mean square of error approximation was less than 0.05, suggesting a satisfactory model (Kline, 1998). Regarding reliability, Cronbach's alpha (internal consistency) greater than 0.80 was superior (George and Mallery, 2010). The average variance extracted (AVE), as well as composite reliability (CR) were greater than 0.70, indicating that the required levels were met (Hair et al., 2014a, Hair et al., 2014b).

To investigate Jordanian's COVID-19 PBB and awareness, means, standard deviations, and overall levels (whether low or high scores calculated in section 2.3) were calculated. Pearson correlation coefficient (*r*) was used to determine the relationship between awareness and PBB. One-way analysis of variance (ANOVA), independent sample t-test, and linear regression (stepwise method) were used to determine the association between PBB and socio-demographics and the association between PBB and awareness. The level of statistical significance was set at *p* ≤ 0.05.

## 3 Results

### 3.1 Demographic characteristics, descriptive analysis, and relationship between the main selected variables

Descriptive statistics of the samples' demographic factors are presented in Table 2. More than half of the participants in this study were female (56.9%, *n* = 244), predominantly married (73.4%, *n* = 315), and had a bachelor's degree (50.3%, *n* = 216). Around 42.7% of the participant had low income (n = 183) from 200 to 500 JD per month (Approximately 282–705 U.S. dollars). The participants age ranged from 18 to more than 65 years old, but the majority of them fall into the range of 26–36 year (43.1 %) (Table 2). The overall perceived level of PBB among the study participants was at low level (1.88 ± 0.47) and the overall perceived awareness level was at high level (3.54 ± 0.37). Results showed that PBB was found to be negatively associated with awareness level (*r* = −.16, *P* < .001). Detailed results for each item in both scales are presented in appendix Table S1.

**Table 2:** Demographic factors and associations with panic buying behavior.

Factors	Descriptive	Frequency	Percentage %	Mean (SD)	t/F	P
Gender	Male	185	43.1	1.87 (0.48)	t = 1.00	.33
	Female	244	56.9	1.89 (0.45)		
Marital status	Single	114	26.6	1.92 (0.52)	t = 4.34	.03∗
	Married	315	73.4	1.87 (0.44)		
Age (Yrs.)	18–25	39	9.1	1.89 (0.47)	F = 2.93	.03∗
	26–36	185	43.1	1.92 (0.49)		
	37–48	130	30.3	1.91 (0.47)		
	More than 48	75	17.5	1.74 (0.35)		
Income per month (J.D.)	200–500	183	42.7	1.88 (0.44)	F = 0.39	.68
	501–1000	155	36.1	1.87 (0.49)		
	More than 1000	91	21.2	1.92 (0.47)		
Educational level	High school	48	11.2	1.87 (0.40)	F = 1.69	.16
	Diploma	64	14.9	1.80 (0.39)		
	Bachelor	216	50.3	1.93 (0.51)		
	Postgraduate	101	23.6	1.84 (0.42)		

*Note. N* = 429. Yrs.: Years; J.D.: Jordanian dinar (200–500 JD is approximately 282–705 U.S. dollars; 501–1000 JD is approximately 706–1410 U.S. dollars); SD: Standard deviation; t: t-test; F: F distribution; ∗*P* < .05.

### 3.2 Measurement results

Before analysis, data were checked and found to be normally distributed, with no multicollinearity, and a total of 71 surveys were excluded due to outlier's data. Regarding PBB scale (13-items) and awareness scale (10-items) content validity, none of the items scored below 0.75 according the arbitrators. Linguistic paraphrasing was done to some items, as arbitrators suggested. The EFA and CFA results for both scales are presented separately as follows:

#### 3.2.1 PBB scale

One factor was loaded with an Eigenvalue of 5.11. The developed PBB scale was responsible for a combined total variation of 42.61%. The KMO test yielded a result of 0.92. Bartlett's test of sphericity was typically significant and suitable for factor analysis of the PBB scale (*χ2* = 1726.01; df = 78; *P* < 0.001). The CFA was measured to determine the cross-loading for each item (13-items), and the results showed that the correlation values ranged from 0.58 to 0.83, except item number nine (0.28), which was deleted. The final version of PBB scale consisted of 12 items. Furthermore, model 1 and model 2 were measured by fit indices (Table 3).

**Table 3:** Fit indices for the CFA model of the PBB scale.

Models	χ2/d.f.	GFI	AGFI	CFI	IFI	TLI	RMSEA
M1	2.85	.93	.91	.93	.93	.91	.06
M2	2.02	.96	.94	.97	.97	.96	.04

*Note. N* = 429. CFA: Confirmatory factor analysis; PBB: Panic-buying behavior; M1: 13 items; M2: 12 items; χ2: Chi square; d.f.: degree of freedom; GFI: Goodness of Fit Index; AGFI: Adjusted Goodness of Fit Index; CFI: Comparative Fit Index; IFI: Increment Fit Index; TLI: Tucker Lewis Index; RMSEA: Root mean square error of approximation.

Table 3 displays the fit indices for models 1 and 2 on the PBB scale. Model 2 (12-items), achieved the required level as follows: relative chi-square was 2.02, which was less than 3; goodness of fit index was 0.96, which indicated perfect model fit; adjusted goodness of fit index was 0.94; comparative fit index value 0.97 was greater than 0.95; increment fit index was 0.97 and Tucker-Lewis index was 0.96, both values were greater than 0.90; root mean square of error approximation was 0.04. Thus, model 2 indicated an acceptable model fit. Regarding reliability, Cronbach's alpha, AVE, and CR achieved the required levels of 0.91, 0.71, and 0.80, respectively.

#### 3.2.2 Awareness scale

The Eigenvalue was 4.24 in one factor and the total variation was 53.87%. The KMO test yielded a result of 0.89, and Bartlett's test of sphericity was found to be typically significant and suitable for factor analysis of awareness scale (*χ2* = 1306.85; df = 45; *p* < 0.001). Correlation coefficients ranged between 0.60–0.87. The following fit indices were obtained for the 10 items: relative chi-square was 2.92; goodness of fit index was 0.92; adjusted goodness of fit index was 0.90; comparative fit index value was 0.95; increment fit index was 0.94; Tucker-Lewis index was 0.94; root mean square of error approximation was 0.05. Each of these results confirmed the model fit. Cronbach's alpha, AVE, and CR, were 0.85, 0.70, 0.68, respectively. These findings indicating the measurement models (PBB and awareness scales) had a good EFA and CFA.

### 3.3 Association of demographic factors with PBB

The t-test was used for gender and marital status (for those with two categories). One-way ANOVA was used with PBB for age, income, and education (for those with three or more categories). Table 2 shows the association of selected demographic factors with PBB. The results indicated that marital status (single) and age (26–36 years old) were associated with increased PBB levels (*p* < 0.05).

### 3.4 Model results

The results of a regression model used to investigate the prediction of PBB with sociodemographic variables, and with awareness of COVID-19 are shown in Table 4. The model highlights that awareness and age predicted PBB with a total variation of 3.1%. Awareness was found to be associated with 1.9% of the PBB variance. Age was also found to be associated with PBB, accounting for 1.2% of the variance in PBB. Other demographic factors were excluded from the model.

**Table 4:** Results of linear regression analysis (stepwise regression) for PBB predictors.

Predictor	Unstandardized Coefficients		r	R^2^	r change	t	95% CI		P
	*b*	*Std. Error*					LL	UL	
Constant	2.492	.214	.137	.019	.019	11.643	2.07	2.91	<.01∗∗
Awareness	−.171	.060				−2.849	−.29	−.05	<.01∗∗
Constant	2.667	.226	.176	.031	.012	11.816	2.22	3.11	<.01∗∗
Awareness	−.178	.060				−2.973	−.29	−.06	<.01∗∗
Age	−.059	.025				−2.339	−.11	−.01	.02∗

*Note. N* = 429. *b*: unstandardized regression weights; *r*: the zero-order correlation; *t*: t-test; *CI* = confidence interval; *LL* = lower limit; *UL* = upper limit; ∗*p <* .05. ∗∗*p <* .01.

## 4 Discussion

This study highlights the perception levels of PBB and awareness of COVID-19. It also provides a newly developed scales of PBB and awareness of COVID-19. Furthermore, an association were found between socio-demographic factors and awareness of COVID-19 with the perceived levels of PBB.

Our first results showed that participants had a low perceived levels of PBB. In particular, the online shopping and the hand delivery emerged as key to limit PBB. With frequent lockdowns, Jordanians trusted that food supply and all essential products are secure and available. This highlights the importance of online shopping to reduce exposure to COVID-19 disease compared to face-to-face shopping. A recent study in Finland citizens revealed that consumers’ behavioral patterns, especially panic-buying, changed dramatically in the first period of the COVID-19 pandemic. Particularly, Finnish citizens expressed extreme panic behavior at the beginning of pandemic which subsided relatively quickly as they shifted to online grocery shopping (Eriksson and Stenius, 2020).

We found that awareness of COVID-19 was perceived to be a critical role for limiting PBB, with a lack of awareness among population recognized as the main challenge. People's awareness is needed to protect themselves during the COVID-19 pandemic, staying at home, keeping a social distance of at least six feet, and adhering to personal protective equipment (Gasmi et al., 2020; Zhong et al., 2020). Safety precautions (e.g. masks, gloves, and hand hygiene) are the best methods for reducing feelings of fear during PBB (Arafat et al., 2021a; Clements, 2020).

The main barrier for obtaining information from reliable sources (government and WHO) is the public distrust of these sources (Harlianty et al., 2020; Kar et al., 2020). Therefore, Government reassurance that the food supply is enough will reduce PBB.

Lower perception of awareness is associated with higher PBB among general population (Jaber et al., 2021), which is in agreement with our findings. Explanations for higher PBB include psychological reactions of COVID-19 disease caused by social media posts (Leung et al., 2021). For example, people will act on what they hear on social media about the impact of COVID-19, which can create more anxiety and fear. Further, assessment of the impact of social media on COVID-19 consequences is still needed.

Surprisingly, single individuals had higher PBB level than married ones. This can be explained by the fact that single individuals struggle with loneliness and do not have someone else to support or to satisfy their needs. Thus, single Jordanians are responsible for obtaining the goods for themselves. In contrast, married people have other family members to support and purchase their needs. Furthermore, age was also found to be associated with PBB. The age group of 26–36 years old showed the highest mean score, which is in agreement a previous study (Li et al., 2021). Potential explanations for the higher PBB in the 26- to 36-year-old more than the other three groups include family consequences, economic situations, and strong relative relationships in the Middle East. However, a study in Singapore found that age variables are not associated with PBB, as younger consumers are considered to be less vulnerable to COVID-19 (Chua et al., 2021). Moreover, Bentall et al. (2021) found that citizens had PBB due the presence of children at home, which were living in the United Kingdom (*N* = 2025) and the Republic of Ireland (*N* = 1041).

The quantitative data in this study suggested that awareness of COVID-19 plays an important role to predict PBB among Jordanians. Jordanians’ awareness of COVID-19 will give control over PBB. Moreover, social distancing and personal protective equipment are very useful in limiting this behavior. Other studies suggested another factors as predictors of PBB such as psychological stress (Zhang and Zhou, 2021) and household income (Bentall et al., 2021).

Finally, new PBB and awareness scales were developed and validated that takes into consideration Jordanian local needs. Those needs were met by having 12 items and 10 items respectively, that were selected based on recent studies, norms, and attitudes among the Jordanian society living in a developing Arab country. However, the Brazilian PBB scale had 7 items specific to their sociodemographic needs which is not necessarily suitable for other developing countries. Content and construct validity by EFA and CFA were in the acceptable ranges, indicating that the developed scales were valid. Similarly, reliability by Cronbach's alpha, CR, and AVE reliability are considered adequate.

Examples of assuring information include those regarding security of food supply, purchasing only the necessary goods, moving to online shopping.

### 4.1 Limitations

Our results are limited by COVID-19 pandemic, sample size, and using online self-reported questionnaires. Other demographic factors such as employment status/living arrangement were not included in this study. However, future studies should focus on these limitations.

## 5 Conclusions and recommendations

Awareness of COVID-19 provides a potential antidote to PBB. Marital status and age factors were found to be associated with PBB. Moreover, we established valid, reliable PBB and awareness scales, and the validity and reliability are achieved at the required levels. However, the validation and reliability of these scales need to be confirmed in different settings and cultures. Individual's COVID-19 awareness must come from trusted sources. Awareness of COVID-19 combined with specific precautions can help reduce threat and anxiety, which lead to PBB. High PBB may affect the world economy, highlighting the importance of COVID-19 awareness. This study will provide scientific and practical benefits for measuring consumer behavior in developing countries. We recommend that policymakers, experts, and researchers provide frequent public educational sessions about COVID-19 and psychological wellbeing to prevent any negative consequences of the pandemic. Further studies on PBB, it's causes, other predictors, and complications are needed to tackle this problem.

## Declarations

### Author contribution statement

Othman A. Alfuqaha: Conceived and designed the experiments; Contributed reagents, materials, analysis tools or data; Wrote the paper.

Dua'a A. Aladwan: Conceived and designed the experiments; Contributed reagents, materials, analysis tools or data.

Yazan Al Thaher; Fadwa N. Alhalaiqa: Performed the experiments; Contributed reagents, materials, analysis tools or data; Wrote the paper.

### Funding statement

This research did not receive any specific grant from funding agencies in the public, commercial, or not-for-profit sectors.

### Data availability statement

Data will be made available on request.

### Declaration of interests statement

The authors declare no conflict of interest.

### Additional information

No additional information is available for this paper.

## Appendix A Supplementary data

Appendix Table S1.docx Appendix Table S1.docx
