# Supplementary material for: Measuring a panic buying behavior: the role of awareness, demographic factors, development, and verification
Source: Heliyon. 2022 May 5;8(5):e09372. doi: 10.1016/j.heliyon.2022.e09372 (PMC9069988; doi:10.1016/j.heliyon.2022.e09372)
Supplement: Appendix Table S1.docx [file mmc1.docx]

**Table S1. Median score results and Interquartile range (IQR) for each question in the developed used scales**

| *#* | *Panic-buying behavior scale* | *Median* | *IQR* |
| --- | --- | --- | --- |
| 1 | When they announced the lockdown, I was anxious about ending food in the supermarket | 2 (Disagree) | 1 |
| 2 | I lost control of buying a huge amount of all home necessities | 2 (Disagree) | 1 |
| 3 | We have never encountered such a situation before: This prompted me to borrow money to buy basic and non-basic needs | 2 (Disagree) | 1 |
| 4 | I purchased basic and non-basic needs to keep myself and my family away from hunger | 2 (Disagree) | 1 |
| 5 | I don’t care about others if I get what I want from foods | 1 (Strongly Disagree) | 1 |
| 6 | Social media is the main reason why I buy a lot of basic and non-basic needs | 2 (Disagree) | 0 |
| 7 | I queued for a long time to meet my basic and non-basic needs | 2 (Disagree) | 1 |
| 8 | The crowded people I saw in the supermarket prompted me to take this behavior | 2 (Disagree) | 1 |
| 9 | My family and I went shopping in groups for fear of running out | 1 (Strongly Disagree) | 1 |
| 10 | The lack of confidence in food reserves and the promulgation of the National Defense Law prompted me to purchase in large quantities | 2 (Disagree) | 1 |
| 11 | The collapse of the global health system in some countries has prompted me to buy a lot of medicines, some of which are what I want and some I don’t | 2 (Disagree) | 1 |
| 12 | The society talks about the spread of COVID-19, prompting me to buy products without considering the shelf life | 2 (Disagree) | 1 |
|  |  |  |  |
| *#* | *Awareness scale* | *Median* | *IQR* |
| 1 | I only rely on official information to raise my awareness of COVID-19 updates | 4 (Strongly Agree) | 1 |
| 2 | I follow the precautionary measures that keep me away from COVID-19 disease | 4 (Strongly Agree) | 1 |
| 3 | The spread of COVID-19 makes me reconsider my social life habits | 3 (Agree) | 1 |
| 4 | The COVID-19 crisis has raised my awareness of the importance of hygiene and several disinfection materials | 4 (Strongly Agree) | 1 |
| 5 | This crisis has a positive side, by raising community awareness, | 4 (Strongly Agree) | 1 |
| 6 | I raised my awareness not to abuse basic and non-basic needs | 4 (Strongly Agree) | 1 |
| 7 | Due to the COVID-19 crisis, I increased my awareness by reducing consumption and saving for the next future | 3 (Agree) | 1 |
| 8 | The policy that followed to manage COVID-19 crisis in Jordan has contributed to keeping me away from social gatherings and maintaining my health and people who around me | 4 (Strongly Agree) | 1 |
| 9 | Government procedures and lockdowns help reduce the infection rate of COVID-19 | 4 (Strongly Agree) | 0 |
| 10 | The general view of society during the spread of COVID-19 made me realize that I should not care about rumors | 4 (Strongly Agree) | 1 |
